# Day‐case artificial urinary sphincter for post‐prostatectomy incontinence: A comparative pilot study

**DOI:** 10.1002/bco2.412

**Published:** 2024-07-25

**Authors:** Konstantinos Kapriniotis, Ioannis Loufopoulos, Richard Nobrega, Anthony Noah, Helena Gresty, Tamsin Greenwell, Jeremy Ockrim

**Affiliations:** ^1^ Department of Urology, University College London Hospital UCLH@ Westmoreland Street London UK

**Keywords:** artificial urinary sphincter, day‐case, outpatient surgery, post‐prostatectomy incontinence

## Abstract

**Objectives:**

Implantation of an artificial urinary sphincter (AUS) to treat post‐prostatectomy incontinence (PPI) has been traditionally offered with an overnight hospital stay. The aim of this prospective, comparative pilot study was to assess the feasibility and outcomes of the AUS procedure in a day‐case setting.

**Patients and methods:**

We included consecutive patients having primary or redo AUS surgery over an 18‐month period. We excluded patients with previous urethral erosion of AUS, urethroplasty or high anaesthetic risk. All patients were offered day‐case surgery. Patients who declined or could not have day‐case surgery for logistical reasons had standard care with overnight stay and formed the control group for the study. Primary outcome was the proportion of successful same day‐discharges in the day‐case group. We also compared baseline characteristics, complications and continence at 1 year post surgery.

**Results:**

Twelve patients consented for day‐case procedure, and 13 patients had standard overnight care. Mean age was 69.5 years (range 58–79). Twenty‐one patients (84%) had primary AUS, whereas 4 (16%) had a redo procedure. There were no significant differences between the groups in baseline demographics. Median number of pads/24 h was 5 in the day‐case group and 4 in the overnight group. Eight of 12 patients (66.7%) in the day‐case group were successfully discharged on the same day. Failed discharges were due to anaesthetic recovery (*n* = 2), high post‐void residuals that resolved spontaneously (*n* = 1) and intraoperative superficial urethral injury (*n* = 1). All patients in the day‐case group and all but one in the standard of care group were socially continent (0–1 pads) at 1 year post procedure.

**Conclusion:**

Day‐case catheter‐free discharge of AUS patients is feasible and safe in selected patients with comparable continence outcomes and complication rates to those with standard overnight stays.

## 1 INTRODUCTION

Post‐prostatectomy incontinence (PPI) is a common complication of prostate surgery with significant psychosocial and financial implications. Despite the advances in minimally invasive prostate cancer surgery, persistent PPI is reported in over 20% of cases in large studies, whereas it is rarer but well reported complication of transurethral surgery for benign prostatic obstruction (BPO).^1^, ^2^

Artificial urinary sphincter (AUS) has been the gold standard treatment for PPI since its introduction in the 1970s.^3^ Depending on the definition of continence used in different studies, success rates approach or exceed 80%.^4^ Common complications include cuff erosion into the urethra, device infection and mechanical failure with recurrence of stress incontinence; and the device has a limited lifespan and usually requires revision surgery within 10–15 years.^5^, ^6^ Previous history of radiotherapy and comorbidities such as diabetes mellitus have been identified as risk factors of complications and inferior continence outcomes.^6^, ^7^

Over the last few decades, there has been an increased tendency to move towards outpatient surgery for selected procedures of moderate complexity. NHS England targets to perform 75% of elective procedures as day‐case surgery, reducing waiting times and bed occupancy to relieve increasing pressures in healthcare systems by and saving resources.^8^ Experience from both urology and other surgical specialties shows that day‐case surgery is associated with non‐inferior outcomes, increased patient satisfaction and reduced healthcare‐related costs in selected patients. Female stress incontinence surgery has been a successful example of this shift in practice.^9^, ^10^

Implantation of AUS has been traditionally performed with an overnight hospital stay in most healthcare systems. As it is a relatively short operation in experienced hands, without significant physiological disturbance, it is not unreasonable to be offered in a day‐case setting with appropriate safety checks in place. To the best of our knowledge, there is little evidence available in the literature to support this practice, with only a single retrospective series reported.^11^ Therefore, the aim of this prospective, comparative pilot study was to assess the feasibility, safety and clinical outcomes of the AUS procedure in a day‐case setting against a similar cohort of patients having AUS implantation following the current standard of care with an overnight stay.

## 2 PATIENTS AND METHODS

We conducted this prospective, comparative, non‐randomised pilot study within a tertiary referral functional and reconstructive urology unit. The study was approved by the hospital's audit department (local audit number: URO 021/01).

We recruited consecutive patients who had been listed for AUS insertion for PPI and were operated over an 18‐month period (May 2021 to December 2022). All patients were given written information about the aims and the content of this pilot study and consented independently for study on a separate enrolment attendance.

Patients of any age who had transurethral, open retropubic, laparoscopic or robotic radical prostatectomy for BPO or prostate cancer complicated by stress urinary incontinence were suitable. We included patients with previous radiotherapy or stable metastatic disease on androgen deprivation therapy. We excluded patients who had previous major urethral surgery (urethroplasty), urological reconstruction or stress incontinence not related to prostate surgery. We also excluded redo AUS cases following infection/erosion and patients who were deemed high risk for day‐case surgery from an anaesthetic perspective based on the recommendation of the pre‐assessment anaesthetic team of the hospital. However, we included redo AUS cases following mechanical failure of device (without urethral complications) or patients who had previous urethral dilatation for stricture and patent urethra at the time of implantation. Finally, all patients participating in the study were required to demonstrate satisfactory understanding of the study process and postoperative instructions, have an escort to collect them from hospital and spend the first night with them, and live close to medical facilities (<1 h) in the event of an emergency (Table 1).

**TABLE 1:** Inclusion and discharge criteria for day‐case surgery.

Inclusion criteria	Discharge criteria
Primary or redo AUS surgery (excluding cases of previous infection/erosion or neurogenic incontinence)	Normal vital signs and adequate mobilisation within a few hours after surgery
Anaesthetic fitness for day‐ case surgery (typically American Society of Anaesthesiologists (ASA) I or II and selected ASA III cases)	Mild or moderate pain controlled with simple oral analgesia
Adequate comprehension of study aims and postop instructions	Bladder emptying efficiency >75% with post‐void residual volume <150 mL
Residence within 1 h from hospital	No signs of immediate surgical complications (e.g., haematoma)
Escort to collect patient from hospital and spend first night with them	

Abbreviations: AUS, artificial urinary sphincter.

All patients who met the inclusion criteria were offered day‐case surgery. The patients who declined day‐case surgery or could not have day‐case surgery for logistical reasons (e.g., no escort to collect them from hospital, residence in long distance from hospital and not possible for the case to be done first on theatre list) had the standard of care treatment with overnight stay and formed the control group for the study.

As per our unit's standard practice, all patients had preoperative video‐cystometrogram (VCMG) with additional assessment of the retrograde leak point pressure (RLPP) to assess for sphincter competency. If there was suspicion of outflow obstruction on VCMG, patients had flexible cystoscopy to exclude urethral stricture prior to the AUS implantation.

The AMS800 device (Boston Scientific, Marlborough, MA, USA) is the only AUS device used in our institution. All procedures in both groups were performed by the same standardised technique. We performed AUS implantation via a transperineal approach and positioned the AUS cuff around the bulbar urethra after splitting the bulbospongiosus muscle. We inserted the pressure‐regulating balloon in the extra peritoneal space via an inguinal incision. A 61–70 cm H_2_O pressure‐regulating balloon was used in all but two cases. We utilised a 71–80 cm H_2_O in two redo cases who had persistent stress incontinence with the standard 61–70 cm H_2_O reservoir. The scrotal pump was positioned in a sub dartos pouch in the dependent ipsilateral scrotum via the same inguinal incision.

A 16 F straight tip Foley catheter was inserted at the beginning of the case. The catheter was then removed at the end of the procedure for the day‐case surgery group and on the first postoperative day for the standard of care group. All patients in both groups received a single dose of gentamicin and co‐amoxiclav on table (or alternative gram‐positive cover for patients allergic to penicillin) and two postoperative doses of co‐amoxiclav. The AUS was activated 6 weeks after the procedure.

Patients on the day‐case group were operated early in the morning (first on the list). All patients were assessed for bladder emptying prior to discharge with standard bladder scanners. Emptying efficiency (the ratio between voided volume and total bladder capacity) >75% with a post‐void residual <150 mL was considered successful, with discharge scheduled for the afternoon of the surgical day.

Both groups were followed with a telephone consultation at 7 days post procedure and with a face‐to‐face appointment to activate the AUS at 6 weeks post procedure. Routine follow up appointments were scheduled at 1 year post surgery.

The primary outcome of the study was the proportion of successful same day‐discharges for the day‐case operations. We also reported complications for the two groups within the first 30 days and 1 year post surgery. Continence was assessed based on self‐reported use of pads (per day) before the procedure and at 1 year post surgery. We classified postoperative continence outcomes as continence (no pads), social continence (one protective pad), improvement (reduced number of pads following AUS) or treatment failure (no change in pads). Finally, baseline demographic, clinical and urodynamics data were recorded and analysed for the two groups of patients.

We decided not to perform a formal statistical analysis due to the small number of patients included in the two groups, but we present descriptive statistics.

## 3 RESULTS

Twenty‐five patients were included in the study. Twelve patients consented for a day‐case procedure, and 13 patients preferred to stay overnight and follow the standard of care pathway. Mean age of the participants was 69.5 years (range 58–79). Twenty‐one patients (84%) had a primary AUS, whereas four patients (16%) had a redo procedure. Twenty‐one patients (84%) had stress urinary incontinence demonstrated on VCMG, whereas three patients (12%) had no SUI demonstrable on video fluoroscopy, and the decision for AUS was based on the clinical demonstration of stress leakage, low RLPP (<60 cm H_2_0) and positive pad test. There were no baseline urodynamic data available for one redo case (4%). Seventeen patients (68%) had a history of robotic prostatectomy, two patients (8%) had previous laparoscopic prostatectomy and two patients (8%) had previous open prostatectomy. Four patients (16%) had stress urinary incontinence (SUI) following transurethral prostate surgery for BPO.

Baseline demographic and urodynamic characteristics of study participants are presented on Tables 2 and 3. Although we did not perform a formal statistical analysis due to the small number of patients included, most of these parameters were similar between the two groups. Median number of pads/24 h was 5 in the day‐case group and 4 in the overnight group. We noted a significantly longer duration of incontinence in the patients operated with the standard of care protocol comparing to the day‐cases (63 vs. 33 months). There were, also, more radiotherapy cases in the overnight stay group (6/13 patients [46.2%] vs. 1/12 patients [8.3%]).

**TABLE 2:** Baseline characteristics of participants.

	Day‐case surgery (*N* = 12)	Overnight stay (*N* = 13)
Age (median [IQR])	71 (6.5)	74 (15.5)
BMI (mean [SD])	27.6 (2.9)	29.6 (5.0)
ASA > 2 (*n* [%])	2 (16.7%)	3 (23.1%)
Smokers	2 (16.7%)	2 (18.2%)
Anticoagulant therapy	0	0
Duration of incontinence (median, [IQR] in months)^a^	36 (23.5)	66 (49)
Redo‐AUS (*n* [%])	1 (8.3%)	3 (23.1%)
Type of prostate surgery (*n* [%])		
Transurethral	3 (25%)	1 (7.7%)
Open	0 (0%)	2 (15.4%)
Laparoscopic/robotic	9 (75%)	10 (77%)
Radiotherapy	1 (8.3%)	6 (46.2%)
Previous urological surgeries (*n* [%])^b^	5 (41.7%)	9 (69.2%)

Abbreviations: AUS, artificial urinary sphincter; IQR, interquartile range.

^a^ For primary cases.

^b^ Urethral dilatation, AUS, male sling, high‐intensity focused ultrasound (HIFU), Botox, transurethral resection of the prostate (TURP).

**TABLE 3:** Urodynamic baseline characteristics (for primary cases).

	Day‐case surgery (*N* = 11)	Overnight stay (*N* = 10)
Capacity (median, IQR in mL)	449 (123.5)	416.5 (199)
Qmax (mean [SD] in mL/s)	16 (6.7)	18 (10.5)
Post void residual (PVR) (median [range] in mL)	0 (0.135)	0 (0)
Stress incontinence (*n* [%])	10 (91%)	9 (90%)
Detrusor overactivity (*n* [%])	4 (36.4%)	4 (40%)
RLPP (mean [SD] in cm H_2_O)	39.6 (11.2)	33.6 (11.1)

Abbreviations: IQR, interquartile range; RLPP, retrograde leak point pressure.

Eight of the 12 patients (66.7%) on the day‐case group were successfully discharged on the same day. The reasons for the failed discharges included postoperative symptomatic bradycardia (*n* = 1), slow recovery from the anaesthetic (*n* = 1), initially high post‐void residuals, which resolved without intervention (*n* = 1), and decision to keep urethral catheter for 48 h due to suspected superficial urethral injury at the time of surgery (*n* = 1).

At the end of the study, all patients in the day‐case group and all but one patient in the overnight stay group were continent or socially continent (0–1 pads/24 h). The only patient who had to use two pads post procedure had residual urgency‐predominant incontinence and was started on pharmacological treatment. Postoperative reduction in the number of pads (median −4 vs. −3 pads) was similar between the two groups of patients (Table 4).

**TABLE 4:** Continence outcomes and complications.

	Day‐case surgery (*N* = 12)	Overnight stay (*N* = 13)
Pads pre‐AUS (median [range])	5 (1, 8)	4 (1, 6)
Pads post‐AUS (median [range])	0.5 (0, 1)	1 (0, 2)
Change in pads (median [range])	4 (1, 7)	3 (1, 6)
Postop complications (Clavien‐Dindo ≥2)	2^a^	1^b^

Abbreviation: AUS, artificial urinary sphincter.

^a^ Clot retention, transient retention requiring suprapubic catheter.

^b^ Infection/erosion.

Regarding postoperative urological complications, one patient in the day‐case group went into urinary retention following removal of urethral catheter in two occasions over 3 days. This patient had the suspected superficial urethral injury during the operation. Eventually, he was managed with a suprapubic catheter in the short term, which was removed successfully 8 weeks after the procedure. At the end of the study, the outcome of his AUS was excellent with no need for pads. In addition, one of the successful day‐cases had clot retention related to radiation cystitis at 4 months post procedure and was managed with bladder washout under general anaesthesia without compromising the excellent outcome of his AUS (no need for pads). This was not considered directly associated with the AUS implant. One patient from the overnight stay group had an infected‐eroded AUS at 6 months post procedure and underwent emergency explanations of the device. He had a radiotherapy‐related stricture before the AUS procedure and was established on self‐dilation. No device explanations were reported in the day‐case group.

## 4 DISCUSSION

This prospective comparative pilot study demonstrated that the AUS procedure is feasible and safe for selected patients in an outpatient setting. Two of the four failed discharges in the day‐case group of patients were related to medical issues or delayed recovery from the anaesthetic. One delayed discharge was related to a urethral injury at the time of AUS cuff implant, who subsequently went on to have protracted voiding dysfunction, even though he was kept in for longer (48 h) observation than standard care. Only one patient had procedure specific issues with higher than protocoled residuals that obligated conversion from day‐case to overnight stay.

None of these discharged (67%) day‐case patients needed intervention to resolve a procedure‐related complication or to be readmitted in the hospital within 30 days of the procedure. In addition, the midterm continence outcomes and overall complications by the end of the study were comparable in the two groups and in keeping with the known outcomes of this procedure published in the literature.^4^, ^5^, ^6^

These conclusions are particularly important in the current climate of increased pressures in healthcare systems and continuing ‘bed crisis’. In high volume centres where >20 AUS procedures are performed every year, a shift to outpatient surgery will free significant capacity for inpatient activity, reduce waiting lists and save money. This has already been shown in various studies for different urological procedures and within different healthcare systems, such as in penile prosthesis surgery in Spain and Italy.^12^, ^13^ The application of telemedicine that has become increasingly popular since the COVID‐19 pandemic has been shown to further support this shift and contributed to a wider acceptance by patients that would otherwise be more reluctant towards outpatient procedures.^14^

To the best of our knowledge, there is only very limited published evidence reporting the outcomes of the AUS procedure in a day‐case setting. Nasri et al. published a retrospective series of 81 primary and revision AUS cases performed in an outpatient setting with similar inclusion criteria to the present study.^11^ However, the majority (65%) were discharged with a catheter in situ, to be removed the next day, and so, this study differed substantially from our one where the outcomes were based on catheter‐free discharge. Nasri et al. reported that 71.6% of their patients were successfully discharged on the day of the operation with only five patients staying overnight or being readmitted within 3 days of the procedure. Thirty‐five minor complications occurred within 90 days of discharge including urinary retention in four (4.9%) of patients, and four major complications occurred (device infection, urethral erosion, reservoir hernia and suprapubic tube placement for urinary retention) within 90 days of surgery, although none of these later complications are necessarily related to the day‐case nature of the intervention.^11^ In addition, Bassi et al. reported 30 AUS procedures (primary or revision cases) performed in an outpatient setting with catheter removal in the end of the case. They reported only one failed discharge, a readmission rate of 6.7% within 30 days of surgery and a removal/revision rate of 6.7%. Social continence was achieved in 88.9% of their patients.^15^ Finally, Dropkin et al. reported a very low perioperative and peri‐discharge complication rate and low opioid requirements post AUS in their retrospective series of 163 AUS cases staying in hospital for one night and therefore suggested that an outpatient procedure could be feasible for selected patients.^16^

The concept of outpatient surgery is not new in urology, and for other inguinoscrotal procedures of similar complexity. Several studies report excellent outcomes of day‐case inflatable penile prosthesis surgery, with similarities to the AUS procedure in terms of length of procedure, size and position of incisions and use of prosthetic implants.^17^ A key difference that has been anecdotally reported as a reason to keep AUS patients overnight is the risk of postoperative retention that comes with a same day catheter removal. However, in this study, none of the patients who had their catheters removed in the end of the procedure (11/12 intended patients) needed re‐catheterization. Similarly, in the study by Nasri et al., 28 of their 81 day‐cases had their catheter removed on the day of the operation with only one of them (3.6%) experiencing acute retention.^11^ Catheter removal in the outpatient department or by the patients at home on the first postoperative day could be valid alternatives in cases deemed to be at higher risk of postoperative retention.

This study is inevitably subject to limitations. Firstly, it is a pilot study, and therefore, the number of patents included was relatively small. In addition, although the baseline characteristics were generally not significantly different between the two groups, the allocation of patients was not randomised, which introduces bias in the interpretation of the results. We gave patients the option to choose between day‐case surgery and standard of care treatment as this was only meant to be a preliminary report to assess a new treatment pathway in our country. However, we feel that this study achieved its main purposes, particularly in demonstrating that day‐case AUS is feasible, and that was the selected (preferred) treatment pathway by almost half of the patients that were considered eligible to have it. The majority of cases in the day‐case cohort would be considered simple AUS cases. We mainly excluded cases post infection/erosion as they had already experienced a treatment‐related complication before. We also excluded cases of neurogenic stress incontinence, as these patients typically have more complex needs that require inpatient treatment immediately after surgery. However, we feel that in the future, we can extend the inclusion to more complex cases that are fit enough from the anaesthetic point of view. Importantly, patients engaged well with the study protocol, and there were no dropouts. This is an important consideration in planning a larger future study to validate our observations. Having reported the first encouraging outcomes here, we continue to offer day‐case AUS surgery and gather data to populate our case series. This is likely to further guide patients' choice towards ambulatory surgery. A multi‐centre randomised prospective study is recommended.

## 5 CONCLUSION

Day‐case implantation of AUS with catheter‐free discharge is feasible and safe in selected patients with comparable continence outcomes and complication rates to those with standard overnight stays. A day‐case approach offers utilisation and financial advantages to heath care providers and may be the preferred option for many patients.

## AUTHOR CONTRIBUTIONS

All authors were involved in and were responsible for the conception, methodology, investigation, data analysis and project administration. Reviewing and editing were performed by Mr Konstantinos Kapriniotis and the senior author Mr Jeremy Ockrim.

## CONFLICT OF INTEREST STATEMENT

The authors declare that they have no conflict of interest or conflicting financial interest.
